# Evaluation of Rapid, Early Warning Approaches to Track Shellfish Toxins Associated with *Dinophysis* and *Alexandrium* Blooms

**DOI:** 10.3390/md16010028

**Published:** 2018-01-13

**Authors:** Theresa K. Hattenrath-Lehmann, Mark W. Lusty, Ryan B. Wallace, Bennie Haynes, Zhihong Wang, Maggie Broadwater, Jonathan R. Deeds, Steve L. Morton, William Hastback, Leonora Porter, Karen Chytalo, Christopher J. Gobler

**Affiliations:** 1 School of Marine and Atmospheric Sciences, Stony Brook University, Southampton, NY 11968, USA; theresa.hattenrath@health.ny.gov (T.K.H.-L.); mark.lusty@stonybrook.edu (M.W.L.); ryan.wallace@stonybrook.edu (R.B.W.); 2 Stressor Detection and Impacts Division, National Centers for Coastal Ocean Science, NOAA National Ocean Service, Charleston, SC 29412, USA; bennie.haynes@noaa.gov (B.H.); maggie.broadwater@noaa.gov (M.B.); steve.morton@noaa.gov (S.L.M.); 3 JHT, Inc., under contract to NOAA, NOAA Charleston Lab, National Centers for Coastal Ocean Science, NOAA National Ocean Service, 219 Fort Johnson Road, Charleston, SC 29412, USA; Zhihong.Wang@noaa.gov; 4 US Food and Drug Administration Center for Food Safety and Applied Nutrition, College Park, MD 20740, USA; Jonathan.Deeds@fda.hhs.gov; 5 New York State Department of Environmental Conservation, Setauket, NY 11733, USA; william.hastback@dec.ny.gov (W.H.); leonora.porter@dec.ny.gov (L.P.); karen.chytalo@dec.ny.gov (K.C.)

**Keywords:** *Alexandrium*, *Dinophysis*, DSP toxins, PSP toxins, resin, SPATT, shellfish monitoring

## Abstract

Marine biotoxin-contaminated seafood has caused thousands of poisonings worldwide this century. Given these threats, there is an increasing need for improved technologies that can be easily integrated into coastal monitoring programs. This study evaluates approaches for monitoring toxins associated with recurrent toxin-producing *Alexandrium* and *Dinophysis* blooms on Long Island, NY, USA, which cause paralytic and diarrhetic shellfish poisoning (PSP and DSP), respectively. Within contrasting locations, the dynamics of pelagic *Alexandrium* and *Dinophysis* cell densities, toxins in plankton, and toxins in deployed blue mussels (*Mytilus edulis*) were compared with passive solid-phase adsorption toxin tracking (SPATT) samplers filled with two types of resin, HP20 and XAD-2. Multiple species of wild shellfish were also collected during *Dinophysis* blooms and used to compare toxin content using two different extraction techniques (single dispersive and double exhaustive) and two different toxin analysis assays (liquid chromatography/mass spectrometry and the protein phosphatase inhibition assay (PP2A)) for the measurement of DSP toxins. DSP toxins measured in the HP20 resin were significantly correlated (*R*^2^ = 0.7–0.9, *p* < 0.001) with total DSP toxins in shellfish, but were detected more than three weeks prior to detection in deployed mussels. Both resins adsorbed measurable levels of PSP toxins, but neither quantitatively tracked *Alexandrium* cell densities, toxicity in plankton or toxins in shellfish. DSP extraction and toxin analysis methods did not differ significantly (*p* > 0.05), were highly correlated (*R*^2^ = 0.98–0.99; *p* < 0.001) and provided complete recovery of DSP toxins from standard reference materials. Blue mussels (*Mytilus edulis*) and ribbed mussels (*Geukensia demissa*) were found to accumulate DSP toxins above federal and international standards (160 ng g^−1^) during *Dinophysis* blooms while Eastern oysters (*Crassostrea virginica*) and soft shell clams (*Mya arenaria*) did not. This study demonstrated that SPATT samplers using HP20 resin coupled with PP2A technology could be used to provide early warning of DSP, but not PSP, events for shellfish management.

## 1. Introduction

The range, frequency and intensity of harmful algal blooms (HABs; [1,2,3]) have increased in recent decades. HABs that synthesize biotoxins (e.g., *Alexandrium*, *Dinophysis*) are a growing societal concern in many coastal regions due to their impacts on human health and the economy [4,5]. Paralytic shellfish poisoning (PSP) and diarrhetic shellfish poisoning (DSP) are globally significant human health syndromes that are caused by ingestion of toxins produced by the dinoflagellates *Alexandrium* (saxitoxin and its congeners) and *Dinophysis* (okadaic acid and dinophysistoxins), respectively [1,6]. Of these poisonings, it was found that DSP was among the most prevalent (>1200 cases), likely because DSP was discovered in the 1970s and monitoring programs are not well established for this human health syndrome. PSP, on the other hand, was discovered in the 1920s, and given that the worst case scenario is death due to respiratory failure, there are many well-established monitoring programs worldwide and, therefore, fewer cases (>400) associated with this human health syndrome [7]. While PSP has occurred on multiple U.S. coastlines for decades [8,9,10,11], it is only within the last decade that DSP has, almost simultaneously, emerged as a threat on the West [12], East [13,14] and Gulf [15,16,17] coasts. Outbreaks of these HABs are often associated with substantial economic losses due to the closure of shellfish beds containing toxic shellfish [18,19,20,21]. In some cases, PSP and DSP are occurring concurrently or in succession [14], although little is known about dual toxin occurrence in shellfish. Given these threats, there is an increasing need for improved monitoring and analytical technologies that can be integrated into biotoxin monitoring programs, as well as a better understanding of the accumulation of toxins in shellfish. 

In 2004, MacKenzie et al. [22] introduced a new monitoring technology called solid phase adsorption toxin tracking (SPATT) that was intended to replace or supplement traditional HAB toxin monitoring strategies such as measuring toxins in shellfish. This technology is based on the premise that toxin-producing algae release their toxins into the water column, which can be subsequently adsorbed by specific resins chosen based on the attributes/properties of the targeted toxin (i.e., lipophilic vs. hydrophilic, etc.). MacKenzie et al. [22] validated the utility of SPATT in a field setting and highlighted a number of advantages including elimination of the matrix effects associated with bivalves, ease of use, integration of toxin levels during a bloom rather than a single time-point ‘grab’ sample [23] and cost-effectiveness. Since then, a number of studies have demonstrated the ability of different resins (i.e., HP20, SP700, SP207) to adsorb and track toxins such as okadaic acid, domoic acid, saxitoxin and microcystin in cultures, spiked samples and in the field [23,24,25,26], with some studies taking an ecosystem approach in regards to monitoring for DSP- or PSP-related toxins [22,25,27,28,29,30]. However, regional-specific assessments are necessary given that toxin composition varies based on strain and/or region, and the ability of SPATT to mimic water column or shellfish toxicity for all toxin congeners is not known. Moreover, no study has compared cell densities of *Alexandrium* and *Dinophysis* to their related toxins in phytoplankton concentrates, SPATT and toxin in indicator bivalves such as blue mussels in North America where both HABs are now a significant public health threat.

Beyond monitoring technologies, analytical approaches that are simple, accurate and cost-effective are imperative for regional biotoxin monitoring programs that often cannot afford to purchase and maintain expensive instrumentation such as an LC/MS or HPLC. Colorimetric methods such as enzyme-linked immunosorbent assays (ELISA) and protein phosphatase inhibition assays (i.e., PP2A) that use less expensive instrumentation (i.e., spectrophotometer), provide high throughput analyses and can generate results in shorter time frames have become increasingly popular for shellfish monitoring programs [22,31]. While LC/MS or HPLC can determine the toxin composition (i.e., concentrations of all toxin congeners) of a sample, this requires individual standards for accurate measurement of toxin congener concentrations. Bioassay kits determine total toxin class concentrations, or target-specific congeners, or structurally similar groups of congeners (i.e., ELISA methods have different cross-reactivities with congeners that are antibody-dependent). Thus, bioassays may over- or under-estimate toxicity depending on congener profiles in a sample matrix and the individual congener toxin potencies and cross-reactivities in the bioassay used for measurement [31,32,33,34]. Further, bioassays such as protein phosphatase inhibition assays, which are considered in vitro tests, are often useful because they provide integrated toxicity scores that measure all toxins in relation to their individual potencies. When developing a shellfish safety monitoring program, it is crucial to assess the competencies of multiple approaches in detecting target toxins or toxin classes. 

While the U.S. Food and Drug Administration (USFDA) sets guidance levels for various toxins in shellfish [35], it is the National Shellfish Sanitation Program (NSSP) that determines which methods are ‘approved’ for the re-opening of closed shellfish beds [36]. The current ‘approved methods’ for re-opening shellfish beds closed due to the presence of PSP [36] are mouse bioassay (MBA), receptor binding assay (RBA) and post-column oxidation high performance liquid chromatography (PCOX HPLC), while LC-MS/MS has just recently been approved by the Interstate Shellfish Sanitation Conference (ISSC) for measuring DSP toxins. Other methods, such as phytoplankton cell counts and rapid test kits, have been used, as appropriate, in state monitoring programs for screening purposes or other limited use capacity.

The goal of this study was to explore and refine methods, technologies and approaches for monitoring PSP and DSP within coastal waters. Given the annual recurrence of PSP- and DSP-producing blooms on Long Island, NY, USA, in recent years [14,32,37], this location was used to compare the dynamics of pelagic *Alexandrium* and *Dinophysis* cells, toxins in phytoplankton concentrates (i.e., the particulate fraction), toxins in deployed blue mussels (*Mytilus edulis*) and toxins in passive SPATT samplers with two types of resin, HP20 and XAD-2. Various shellfish species were also collected from local shellfish beds during high-density blooms and used to compare two different extraction techniques (single dispersive vs. double exhaustive) and two different toxin analysis assays (LC/MS vs. PP2A). To our knowledge, this is the first study to concurrently examine the accumulation of both PSP and DSP in SPATT, the first study to assess the causative organisms of PSP and DSP (*Alexandrium* and *Dinophysis*, respectively), the PSP and DSP toxins within those causative organisms and the accumulation of these toxins in shellfish. This is also the first study to quantify DSP toxins (and their esters) and multiple pectenotoxins in shellfish, resin and in *Dinophysis* and to compare those to *Dinophysis* densities, as well as the first study that compares *Alexandrium* cells, toxins in *Alexandrium*, PSP toxins in SPATT and PSP toxins in shellfish. Collectively, the results provide insight useful for HAB and shellfish monitoring programs.

## 2. Materials and Methods

### 2.1. Time Series Field Sampling 

**Figure 1:**
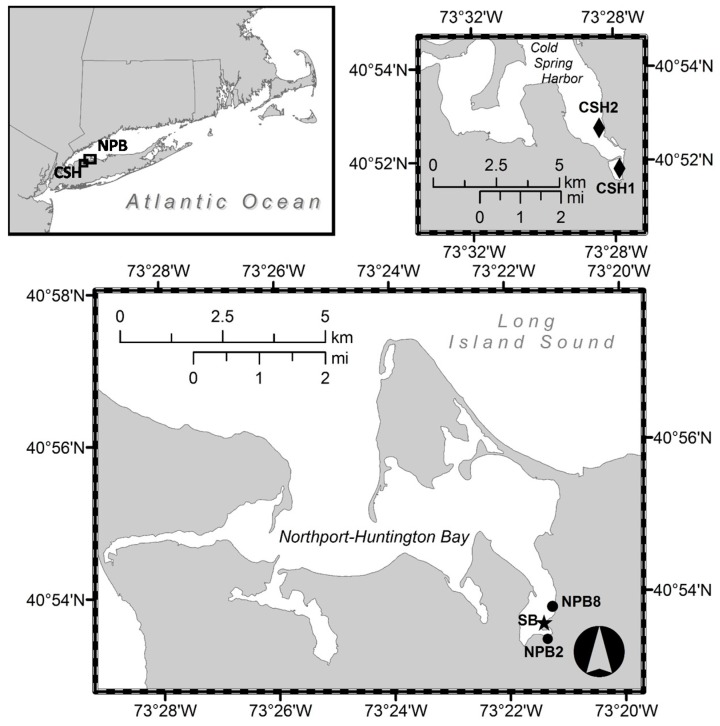
Locations of sampling sites in Northport Bay and Cold Spring Harbor (CSH), New York. Black diamonds = wild shellfish sampling sites in Cold Spring Harbor; black circles = time series sampling sites in Northport Harbor (NPB2 and NPB8), Black star = wild shellfish sampling site Scudder Beach (SB) Northport Harbor.

During 2012, subsurface water (~0.25 m) was collected on a weekly to twice-weekly basis from March–August at two sites, Britannia (NPB2) and Woodbine (NPB8), in Northport Harbor, which is within the southeastern portion of the Northport-Huntington Bay complex, located on the north shore of Long Island, NY, USA (Figure 1). Additional sampling was conducted in 2013 and 2014 at NPB2 and two sites (CSH1 and CSH2) in Cold Spring Harbor, NY, USA (Figure 1). Concentrates were made to increase the limit of detection for cells and toxins as *Dinophysis acuminata* is often a relatively small portion of the total phytoplankton community [14]. Concentrated water samples were made in the field by sieving 1 L of seawater through a 200-µm mesh (to eliminate large zooplankton) and then onto a 20-µm sieve that was backwashed into a 15-mL centrifuge tube. *Dinophysis* cell densities in Lugol’s iodine preserved concentrates (*n* = 2 per sampling date) were enumerated using a 1-mL Sedgewick-Rafter slide. *Alexandrium fundyense* (now *A. catenella* [38]) cell densities were enumerated using a highly sensitive molecular probe developed by Anderson et al. [39] and described at length in Hattenrath et al. [32]. Briefly, duplicate aliquots of phytoplankton concentrates (formalin and then methanol preserved) were hybridized with an oligonucleotide probe specific for the NA1 North American (Group I, recently revised as *A. fundyense* [40]) ribotype *Alexandrium fundyense*/*catenella*/*tamarense* with Cy3 dye conjugated to the 5′ end (5′-/5Cy3/AGT GCA ACA CTC CCA CCA-3′). Cells were enumerated using a Nikon epifluorescence microscope with a Cy3™ filter set [39]. Additionally, cell pellets of phytoplankton concentrates were prepared for toxin analysis by pre-sieving several liters of water through a 200-µm mesh (to eliminate large zooplankton) and subsequently concentrating this biomass on a 20-µm sieve and backwashing biomass into 15-mL centrifuge tubes. Samples were centrifuged at 3000 rpm for 11 min and the supernatant aspirated without disturbing the cell pellet. Cell pellets were kept frozen at −20 °C until further analysis.

### 2.2. Shellfish and SPATT Sampler Deployment and Collection

In the spring of 2012, Vexar mesh bags with ~10-mm openings containing the blue mussel, *Mytilus edulis*, and SPATT (solid-phase adsorption toxin tracking) samplers were deployed in the Northport-Huntington Bay complex (NPB2, NPB8; Figure 1). To construct mussel bags, hundreds of blue mussels (30–50 mm) were collected from Stony Brook Harbor, NY, USA, where PSP and DSP have never been detected (Hattenrath-Lehmann, Gobler, New York State Department of Environmental Conservation). Prior to constructing bags, mussels were tested for the presence of PSP toxins using the methods described below. Fifteen mesh bags containing 15–20 mussels per bag were deployed at two sites (NPB2 and NPB8) in Northport Bay in late March 2012. Mussel bags were collected weekly from April–July at each site, and all mussels were shucked, homogenized and frozen (−20 °C) until analysis. In addition, SPATT samplers were deployed in close proximity to the deployed mussel bags to assess the ability of these devices to track both DSP and PSP toxins. SPATT samplers were constructed from 95-µm nylon mesh, 3-inch embroidery hoops and 3 g (dry weight) of activated resin. Two SPATT devices filled with two different resins, HP20 (Diaion^®^) and XAD-2 (Amberlite^®^), were deployed to compare their abilities to track each toxin (okadaic acid and saxitoxin). Resin was activated according to the manufacturer’s instructions. Briefly, resin was soaked in 100% methanol, rinsed thoroughly with de-ionized (DI) water and stored at 4 °C in DI water [22]. The resin-containing vessels were collected and replaced weekly and frozen at −20 °C until analysis. Finally, in 2013 and 2014, native shellfish including soft shell clams (*Mya arenaria*), ribbed mussels (*Geukensia demissa*), blue mussels (*Mytilus edulis*) and Eastern oysters (*Crassostrea virginica*) were harvested from Northport Harbor (Scudder Beach, SB, star, Figure 1) and Cold Spring Harbor (CSH1, CSH2, diamonds, Figure 1), shucked, homogenized and frozen (−20 °C) until analysis. Homogenate for the sample was prepared from at least 6 pooled individuals of the same species. 

### 2.3. Analysis of PSP Toxins in Algal Pellets, Shellfish and SPATT

The algal pellets were extracted in 0.1 N HCl in 15-mL polypropylene centrifuge tubes (United Laboratory Plastics, St. Louis, MO, USA). The samples were probe sonicated (Branson 1450 sonicator, Thomas Scientific, Swedesboro, NJ, USA) on ice for 2 min and then were placed in boiling water for 7 min. The samples were centrifuged at 1950 *g* for 5 min, and the supernatant for each sample was filtered with a 0.45-µm syringe filter and stored in 8-mL vial at 4 °C until analysis. The SPATT bags were extracted with 10 mL 80% acetonitrile containing 0.1% formic acid in 50-mL polypropylene centrifuge tubes (United Laboratory Plastics, St. Louis, MO, USA). Sample tubes were placed in a beaker containing room temperature water in a sonicator bath for 40 min. Samples were then transferred to clean 50-mL centrifuge tubes and were centrifuged for 10 min at 1950 *g*. The supernatant was filtered with a 0.45-µm syringe filter and stored in 8-mL vials at 4 °C until analysis. Mussel homogenate created from pooled individuals was extracted by the New York State Department of Environmental Conservation (NYSDEC) using standard techniques [41]. 

All samples were screened using a receptor binding assay (RBA) according to standard methodologies [42]. Samples positive for PSP toxins via RBA were analyzed by LC/MS. For algal samples, extracts were diluted 20-fold with 75% acetonitrile/water containing 0.1% acetic acid for LC/MS analysis. For shellfish samples, the extracts were cleaned with C18 solid phase extraction (SPE) cartridges (500 mg in a 3-mL tube, Supelco) and then with graphitized polymer carbon ENVI-Carb SPE cartridges (250 mg in a 3-mL tube, Supelco). The C18 SPE cleanup procedure was adopted from Lawrence et al. [43] with loading of 1 mL extract and the final SPE eluate volume made up to 4 mL water. Next, 1 mL of C18 SPE eluate was added to 2.3 µL of 29% ammonia. Additional ammonia was added until a pH between 6 and 7 was achieved. Next, 400 µL of the above solution were loaded on the conditioned carbon SPE using the procedure described by Boundy et al. [44] for cleanup. After mixing the SPE eluent (2 mL), 100 µL of the SPE eluent were added to 300 µL of acetonitrile for analysis of PSP toxins by liquid chromatography/mass spectrometry (LC/MS). Analyses were performed using an Agilent LC 1100 coupled to a Sciex 4000 QTRAP (Thomas Scientific, Swedesboro, NJ, USA) hybrid triple quadrupole/linear ion trap mass spectrometer equipped with a Turbo VTM source (Sciex, Foster City, CA, USA). PSP toxins were screened using the multiple reaction monitoring (MRM) method with separation of toxins using a TSK-gel Amide-80 column (250 × 2 mm, 5 µm, TOSOH Bioscience LLC, King of Prussia, PA, USA) with 3-µL injections. The mobile phase consisted of 2 mM ammonium formate and 0.1% formic acid in water (A) and 0.1% formic acid in acetonitrile (B) with the following LC gradient: 1 min of 70% B, linear gradient to 30% B at 35 min, held at 30% B for 2 min, linear gradient back to 70% B in 1 min and held at 70% B for 14 min before next injection. The LC flow rate was 0.2 mL min^−1^. A 2-position diverter valve (VICI, Valco instruments CO. Inc., Houston, TX, USA) was used to send the LC eluant to waste except for the time window bracketing all the toxins to MS. All PSP toxins were monitored in positive ion mode with MRM channel settings similar to those described by Boundy et al. [44]; negative ion mode was not used in combination with the positive ion mode due to the limitation of 4000QTRAP. The MRM transitions were listed along with their collision energy (CE) in the unit of ev: *m*/*z* 257.1→126.1 (CE 29) and 239.1 (CE 25) for dcSTX, *m*/*z* 273.1→126.1 (CE 33) and 255.1 (CE 20) for dcNEO, *m*/*z* 300.1→204.1 (CE 33) and 282.1 (CE 27) for STX, *m*/*z* 316.1→220.1 (CE 33) and 298.1 (CE 27) for NEO and M2, *m*/*z* 273.1→126.1 and *m*/*z* 353.1→273.1 (CE 17) for dcGTX2, *m*/*z* 353.1→255.1 (CE 27) and 273.1 for dcGTX3, *m*/*z* 316.1→298.1 and *m*/*z* 396.1→316.1 (CE 16) for GTX2, *m*/*z* 396.1→298.1 (CE 28) and 316.1 for GTX3, C1, C2, M1, and M5, *m*/*z* 332.1→314.1 (CE 28), *m*/*z* 412.1→332.1 (CE 20) for GTX1, *m*/*z* 412.1→314.1 (CE 28) and 332.1 for C3, C4, GTX4 and M3 and *m*/*z* 380.1→282.1 (CE 31) and 300.1 (CE 21) for GTX5. PSP toxins were quantified against National Research Council of Canada (NRC) certified PSP toxin standards when toxin standards were available or against their structural similar congeners as described in Boundy et al. [44] when toxin standards were not available. Each toxin concentration was converted to saxitoxin•2HCl equivalent using toxicity equivalency factors (TEF) edited by Turner et al. [45] and combined. The limits of detection (LOD; in nM) were approximately 54.2 for dcNEO, 1.1 for STX, 4.1 for NEO, 3.9 for dcGTX2, 1.1 for dcGTX3, 5.5 for GTX2, 2.8 for GTX3, 9.1 for GTX1, 0.9 for GTX4, 4.5 for GTX5, 3.5 for C1 and 1.0 for C2, for the toxin standards in the solvent (75% acetonitrile/water containing 0.1% acetic acid) with the signal-to-noise ratio of the corresponding MRM confirmation channel about 3. The limits of quantitation (LOQ; in nM) were 54.2 for dcNEO, 1.7 for STX, 8.4 for NEO, 19 for dcGTX2, 3.1 for dcGTX3, 49.2 for GTX2, 2.8 for GTX3 22.9 for GTX1, 0.9 for GTX4, 4.5 for GTX5, 3.5 for C1 and 1.2 for C2, for the toxin standards in the solvent with the signal-to-noise ratio of the corresponding MRM quantitation channel about 10 or above. The LOD and LOQ were much higher for each PSP toxin in samples than in the solvent for calibration purposes, which could be estimated from the sample dilution factors and clean-up procedure described above. The sample dilution and clean-up procedure were necessary in order to reduce the sample matrix effect for toxin detection against toxin standards prepared in solvents without sample matrix. Toxins M1, M2, M3 and M5 were detected in shellfish and in total accounted for 40–50% of the total toxicity; however, the quantitation of those M toxins may have large errors due to the lack of M toxin standards. Selected samples that were positive for PSP toxins by the MRM method were further analyzed using their product ion spectra to confirm their presence. In addition, LC/MS data were compared to toxin levels in shellfish that were quantified using standard mouse bioassays [41] performed by National Shellfish Sanitation Program (NSSP) evaluated laboratories (NYSDEC lab and Resource Access International, LLC, Brunswick, ME, USA).

### 2.4. Analysis of DSP Toxins in Algal Pellets, Shellfish and SPATT

Algal pellets were resuspended in 5 mL of 80% aqueous methanol, homogenized by vortex mixing and probe-sonicated (Branson 1450 sonicator) on ice at 30% amplitude, followed by centrifugation at 1950 *g* for 10 min. Shellfish homogenate (approximately 2 g of 15–20 pooled individuals for blue mussels and at least 6 pooled individuals for all other species) was extracted in four volumes of 100% methanol, followed by centrifugation at 1950 *g* for 5 min. Methanolic supernatants from algal pellets and shellfish were filtered with a 0.45-μm syringe filter in preparation for analysis. SPATT bags were soaked 2× in 500 mL of DI water for 5 min. The SPATT bag was then soaked in 80 mL of 100% methanol for 2 h and the supernatant poured into a round bottom flask. A second 80 mL of 100% methanol was added to the beaker containing the SPATT bag. It was swirled and placed into sonicator bath for 1 min. Both extractions were combined in the round bottom flask and rotary evaporated to dryness in a water bath at 50 °C. The round bottom flask was rinsed with 15 mL of 100% methanol and transferred to a glass tube that was placed into a turbo evaporator and evaporated with nitrogen to dryness at 40 °C. This was then resuspended in 5 mL of 80% methanol and filtered with a 0.45-μm syringe filter and stored at −20 °C until analysis. 

Samples were analyzed for the presence of DSP toxins and pectenotoxins using liquid chromatography (HP 1100) coupled with tandem mass spectrometry (4000QTRAP, AB Sciex Pte. Ltd., Framingham, MA, USA) using the method described by [46,47] with modifications. LC separation was performed on X-BridgeTM C18 (150 × 3 mm, 5 µm) column (Waters, Milford, MA, USA) using a mobile phase of water (A) and acetonitrile/water (90:10, *v*/*v*) (B), both containing 6.7 mM ammonium hydroxide under gradient elution at a flow rate of 0.4 mL min^−1^. The LC gradient was: 3.5 min of 10% B, linear gradient to 90% B at 14.5 min, held for 3 min, return to 10% B at 19.5 min and held for 4 min. The same as the analysis of PSP toxins, the 2-position diverter valve functioned to send LC eluant between waste and MS. In order to maintain the MS stability for sensitivity, LC gradient programming was designed in combination with the diverter valve by adding more 10% B (e.g., 4.5 or 5 min instead of 3.5 min of 10% B at initial gradient) and about 1.5 min 100% B after 90% B to wash out salt and hydrophobic matrix injected from hydrolyzed SPATT samples to waste. For hydrolyzed algal pellet samples, also more 10% B washing of LC column and a slow LC gradient were used to reduce the matrix effect: 4 min of 10% B, linear gradient to 23% B at 4.5 min and to 48% B at 15 min, linear gradient to 90% B at 19 min and held for 3 min, return to 10% B at 20.5 min and held for 4.5 min. The toxin detection by MS was achieved by multiple reaction monitoring (MRM) in negative ion mode for OA, DTX1 and DTX2 (for OA and DTX2 with MRM transitions of *m*/*z* 803.5→113.1 and 255.1, for DTX1 with MRM transitions of *m*/*z* 817.5→113.1 and 255.1, with CE −85 and −67 ev for MRM confirmation channel and quantitation channel, respectively) and in positive ion mode for PTX1, PTX11, PTX2 and their isomers (for PTX1, PTX11 and their isomers with MRM transitions of *m*/*z* 892.5→213.1 and 839.5, for PTX2 and its isomers with MRM transitions of *m*/*z* 876.5→213.1 and 823.5, with CE 55 and 35 ev for MRM confirmation channel and quantitation channel, respectively). Certified standards of OA, DTX1, DTX2, and PTX2 were available for toxin determination from NRC (Halifax, NS, Canada). No certified standards are available for PTX1, PTX11 and their isomers and PTX2 isomers; their concentrations were calculated approximately using PTX2 standards. PTX1, PTX11 and their isomers could not be clearly identified with their product ion spectra due to their low concentration; one isomer carried more characteristic fragments of PTX11 [48] in its product ion spectra (enhanced product ion spectrum with the linear ion trap function and MS2 product ion spectrum with triple quadrupole function). Therefore, PTX11-like compounds were used to express PTX11, PTX1 and other PTX isomers in this report because they had the same molecular mass, and their product ion spectra acquired from the samples showed the same major fragments. PTX2 and its isomers also shared the same molecular mass and same major fragments in product ion spectra, but different LC retention time. As such, all PTX concentrations were combined and reported as total PTXs (herein referred to as PTX). In addition to analyzing free DSP toxins, samples were subjected to alkaline hydrolysis for the determination of esterified DSP toxins. One hundred fifty microliters of 2.5 M sodium hydroxide solution were added to 1.2 mL of sample solution (for shellfish samples, 1.2 mL sample solution contained 0.12 g tissue equivalent extract). The mixture was placed in a water bath at 76 °C for 45 min, allowed to cool to room temperature and then neutralized with a 150 µL of 2.5 M hydrochloric acid solution [49]. The sensitivity of LC/MS varied slightly with samples and day-to-day runs. The LODs were about 0.08, 0.08, 0.08 and 0.04 ng/mL for OA, DTX2, DTX1 and PTX2, respectively, for toxin standards in methanol for monitoring cell pellets and SPATT bag samples and were about 3, 3, 4 and 0.8 ng/g for OA, DTX2, DTX1 and PTX2, respectively, for monitoring the shellfish samples with calibration standards prepared in shellfish matrix, with the signal-to-noise ratio of 3 or higher for the corresponding MRM confirmation channels. The LOQs were about 0.08, 0.08, 0.18 and 0.05 ng/mL for OA, DTX2, DTX1 and PTX2, respectively, for toxin standards in methanol for monitoring the cell pellets and SPATT bags samples and were about 5, 5, 8 and 1.1 ng/g for OA, DTX2, DTX1 and PTX2, respectively, for the shellfish samples, with the signal-to-noise ratio of 10 or higher for the corresponding MRM quantitation channels, with a 5-µL injection volume.

### 2.5. Comparison of DSP Toxin Analysis Methodologies, LC/MS and PP2A

In addition to analyzing DSP toxins via LC/MS, a colorimetric protein phosphatase inhibition assay (PP2A) for quantifying total DSP toxins (okadaic acid and DTX’s) was utilized. DSP-specific PP2A kits (Okadaic acid, cat# PN 520025) were purchased from Abraxis©, and shellfish homogenate was extracted, hydrolyzed and analyzed according to the manufacturer’s protocol. In addition to using the suggested Abraxis© extraction procedures (single dispersive), the kit was further tested using extraction procedures recommended by the European Union (double exhaustive; [50]) for the measurement of lipophilic toxins by LC-MS/MS. These comparisons were made to streamline extraction procedures so the same extraction could be used to screen samples using PP2A and then confirmed, when necessary, by LC-MS/MS. Briefly, the Abraxis© extraction procedure calls for extracting 5 g of shellfish homogenate in 25 mL of 100% methanol (1:5, tissue:methanol), whereas the European Union extracts 2 g of shellfish homogenate twice in 9 mL of methanol, combines the supernatant and brings this up to 20 mL of 100% methanol (1:10, tissue:methanol). Further, the PP2A Abraxis© kit was validated using certified reference materials purchased from the National Research Council of Canada (NRC). Certified reference materials included a non-toxic mussel homogenate (CRM-zero-mus), a naturally-contaminated mussel sample (CRM-DSP-mus-c, not hydrolyzed) analyzed via LC/MS (total toxicity (free toxins) = 3000 ng g^−1^) and a methanolic calibration standard (CRM-OA-c, 17 µM) diluted with methanol to fit within the range of the assay curve (1.5 nM). Both extraction procedures and analysis methods (PP2A vs. LC/MS) were compared across multiple samples.

### 2.6. Statistical Analysis

Student *t*-tests were used to compare among the two extraction methods (single dispersive vs. double exhaustive), as well as the DSP analysis methods (PP2A vs. LC/MS). In addition, linear regression analyses were used to examine the relationships among *Dinophysis* densities and toxins in *Dinophysis* cells, shellfish and resin in SPATT samplers. Linear regressions were also used to examine the relationship between DSP analysis methods (PP2A vs. LC/MS) for several species of shellfish. All statistical analyses were conducted using Sigma Plot 11.0 (Systat Software, Inc., San Jose, CA, USA).

## 3. Results

### 3.1. SPATT Technology: Comparison of HP20 and XAD2 Resins

**Figure 2:**
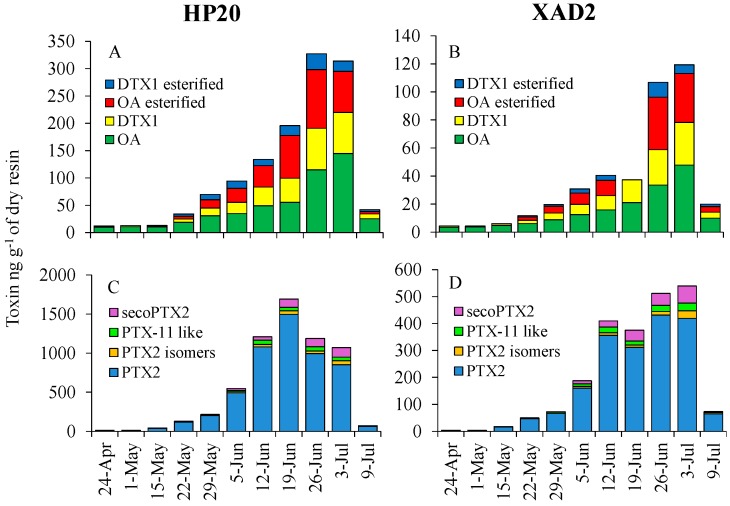
Comparison of (**A**,**B**) DSP toxins (OA, DTX1, esterified OA and esterified DTX1; ng g^−1^ of dry resin) and (**C**,**D**) pectenotoxins (PTX2, PTX2 isomers, secoPTX2 and PTX-11-like compounds) adsorbed by two resins (HP20 and XAD-2) in passive solid-phase adsorption toxin tracking (SPATT) samplers deployed side-by-side at NPB2 (Britannia) in Northport Harbor, New York. Bars represent the value of a single sample.

**Figure 3:**
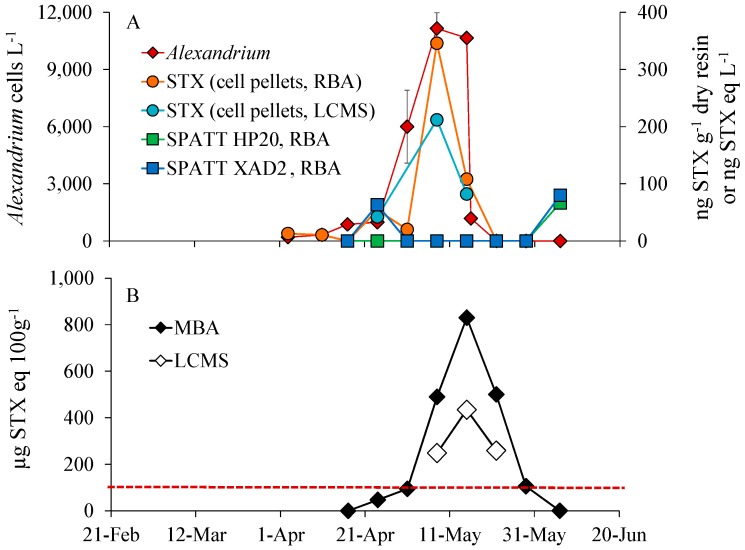
(**A**) Dynamics of *Alexandrium* (cells L^−1^), PSP toxins in phytoplankton concentrates (measured via receptor binding assay (RBA) and LC/MS; ng STX eq L^−1^) and PSP toxins adsorbed by SPATT resins HP20 and XAD-2 (measured via RBA; ng STX g^−1^ dry resin). Points are means, while error bars represent the standard deviation of duplicate samples. (**B**) PSP (µg STX eq 100 g^−1^) in blue mussels, hung in Woodbine (NPB8) Northport Harbor, NY, measured via mouse bioassay (MBA), and liquid chromatography mass spectrometry (LC/MS). Points represent the value of a single toxin sample analyzed from 15–20 pooled individuals.

HP20 and XAD-2 resins were capable of adsorbing multiple toxins associated with *Dinophysis*, including DSP toxins (OA, DTX and their esters; Figure 2A,B) and pectenotoxins (secoPTX2, PTX2, isomers of PTX2 and PTX11-like toxins; Figure 2C,D). Total DSP concentration ranges were 11.9–327 and 4.3–120 ng g^−1^ dry resin for HP20 and XAD-2 resins, respectively, while total PTX ranges were 11.2–1692 and 3.7–539 ng g^−1^ dry resin for HP20 and XAD-2 resins, respectively (Figure 2). Among the DSP toxins, OA, DTX1, esterified OA and esterified DTX1 represented 53%, 20%, 19% and 8%, respectively, of total DSP toxins adsorbed by the HP20 resins with XAD-2 resins displaying a similar pattern (Figure 2A,B). Among the pectenotoxins, PTX2, PTX2 isomers, PTX11-like and secoPTX2 represented 88%, 5%, 4% and 3%, respectively, of total PTXs adsorbed by the HP20 resins with XAD-2 displaying a similar distribution (Figure 2C,D). On average, the HP20 resin adsorbed 3.1-times more DSP toxins and 2.7-times more PTXs compared with the XAD-2 resin (Figure 2), demonstrating that HP20 resin has a higher absorptive capacity for these toxins. Both resins were capable of adsorbing measurable levels of PSP toxins with PSP toxins detected (via RBA) from extracted SPATT resins at concentrations of 63–80 ng STX g^−1^ dry resin on only three occasions (Figure 3A). 

### 3.2. Alexandrium Bloom Dynamics and PSP Toxins in Phytoplankton Concentrates, Shellfish and SPATT 

The *Alexandrium* bloom peaked at ~11,000 cells L^−1^ (8 May, site NPB8) with maximal particulate toxin concentrations measured via receptor binding assay (RBA) of 346 ng STX eq L^−1^ and 212 ng STX eq L^−1^ as measured via LC/MS (Figure 3A). Shellfish toxicity tracked both *Alexandrium* densities and water column toxicity with shellfish toxicity generally being a week later (Figure 3B). Toxins in blue mussels measured via the mouse bioassay (MBA; [41]) exceeded (94 µg STX eq 100 g^−1^) the federal closure limit (80 µg STX eq 100 g^−1^) on 1 May when cell densities were ~6000 cells L^−1^, peaked on 15 May at 830 µg STX eq 100 g^−1^ of shellfish tissue one week after the bloom peak and remained over the closure limit for over a month (Figure 3). While toxin concentrations measured via LC/MS tracked those measured via MBA, they were 48–50% lower (Figure 3B). Comparing SPATT dynamics to those of the *Alexandrium* bloom, water column toxicity and shellfish toxicity, peak concentrations for HP20 and XAD-2 resins (66 ng STX g^−1^ dry resin measured via RBA) occurred well after the decline of the *Alexandrium* bloom (6 June; Figure 3B). Toxin concentrations in SPATT samplers measured via LC/MS were all below the detection limit of the machine. Neither resin tracked bloom dynamics, planktonic toxicity or shellfish toxicity.

### 3.3. Dinophysis Bloom Dynamics and DSP Toxins in Phytoplankton Concentrates, Shellfish and SPATT 

**Figure 4:**
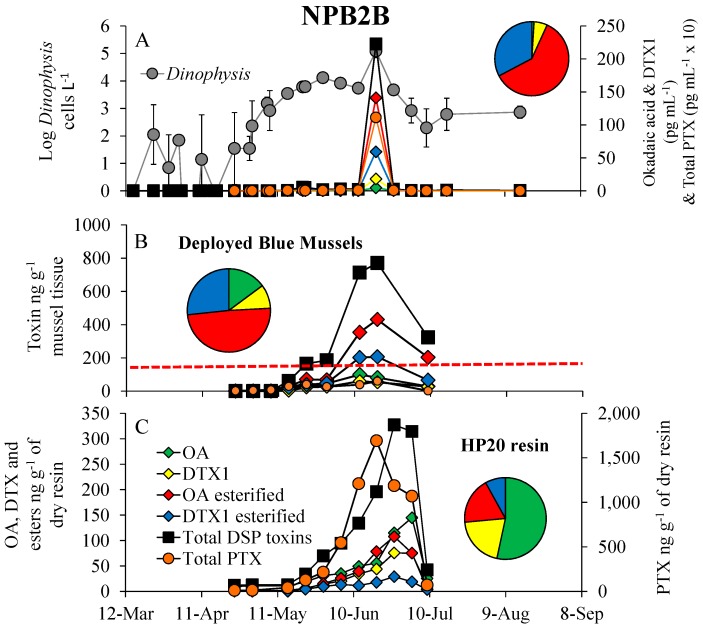
(**A**) Log *Dinophysis* densities (cells L^−1^), the DSP toxins, okadaic acid (OA) dinophysistoxin 1 (DTX1) and their esters (pg mL^−1^) and total pectenotoxins (PTX; pg mL^−1^ × 10) for NPB2 (Britannia) located in Northport Harbor, during 2012. (**B**) Dynamics of the DSP toxins, OA, DTX1 and their esters, as well as total pectenotoxins (ng g^−1^) in blue mussels (*Mytilus edulis*) hung for monitoring purposes. The red line indicates USFDA action level (160 ng g^−1^ shellfish tissue). (**C**) DSP toxins (OA, DTX1 and esters) and pectenotoxins (ng g^−1^ of dry resin) adsorbed by SPATT samplers containing HP20 resin that were hung alongside mussels bags in Northport Harbor. Pie chart insets represent the percent of total toxin each DSP congener represents (colors as in bottom panel). For *Dinophysis* densities, points are means while error bars represent the standard deviation of duplicate samples. For all toxin samples, points represent the value of a single sample. For shellfish, points represent 15–20 individuals pooled prior to toxin analysis.

**Figure 5:**
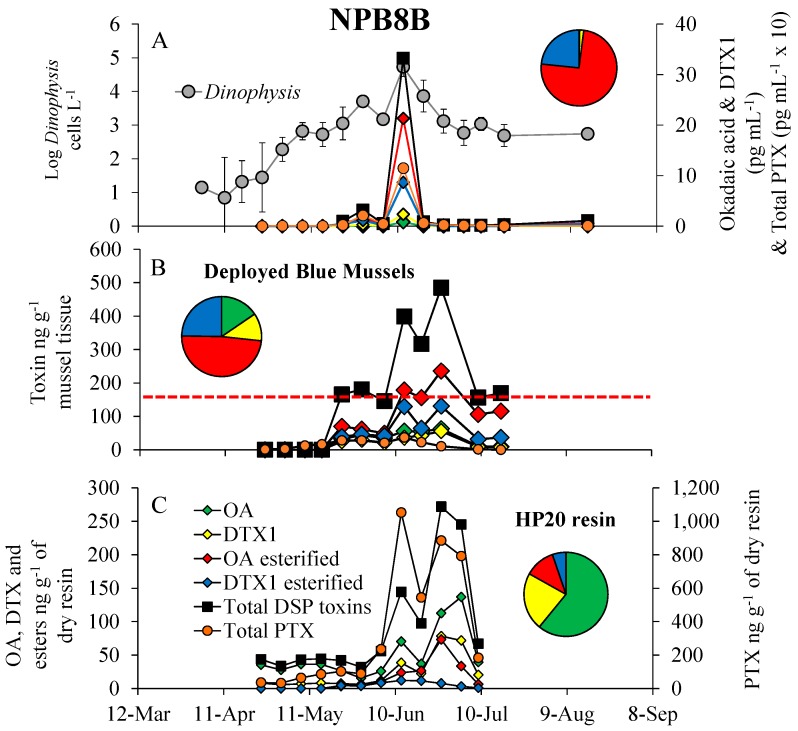
(**A**) Log *Dinophysis* densities (cells L^−1^), the DSP toxins, okadaic acid (OA) dinophysistoxin 1 (DTX1) and their esters (pg mL^−1^) and total pectenotoxins (PTX; pg mL^−1^ × 10) for NPB8 (Woodbine) located in Northport Harbor, during 2012. (**B**) Dynamics of the DSP toxins, OA, DTX1 and their esters, as well as total pectenotoxins (ng g^−1^) in blue mussels (*Mytilus edulis*) hung for monitoring purposes. The red line indicates USFDA action level (160 ng g^−1^ shellfish tissue). (**C**) DSP toxins (OA, DTX1 and esters) and pectenotoxins (ng g^−1^ of dry resin) adsorbed by SPATT samplers containing HP20 resin that were hung alongside mussel bags in Northport Harbor. Pie chart insets represent the percent of total toxin each DSP congener represents (colors as in the bottom panel). For *Dinophysis* densities, points are means, while error bars represent the standard deviation of duplicate samples. For all toxin samples, points represent the value of a single sample. For shellfish, points represent 15–20 individuals pooled prior to toxin analysis.

DSP toxins (OA, DTX1) and their esters and pectenotoxins (PTX) were found in phytoplankton concentrates, blue mussels and HP20 resin in SPATT samplers during the summer of 2012 (Figure 4 and Figure 5). The *Dinophysis* bloom began on 7 May when cell densities first exceeded 1000 cells L^−1^, peaked at 123,000 cells L^−1^ (19 June) and 54,000 cells L^−1^ (12-June) for sites NPB2 and NPB8, respectively, and declined to <1000 cells L^−1^ by 9 July (Figure 4A and Figure 5A). Maximal particulate toxin concentrations were 1110 and 115 pg mL^−1^ for total PTX, and 223 and 33 pg mL^−1^ for total DSP toxins for sites NPB2 and NPB8, respectively (Figure 4A and Figure 5A). PTX concentrations were usually the most abundant particulate toxin with OA esters, DTX1 esters, DTX1 and OA representing 60–75%, 23–33%, 2–6% and 1% of total DSP toxins found in phytoplankton concentrates (i.e., the particulate fraction) with peak toxin concentrations coinciding with the peak *Dinophysis* densities (Figure 4A and Figure 5A). Shellfish toxicity also generally tracked both *Dinophysis* densities and toxicity (Figure 4A,B and Figure 5A,B). In contrast to particulate toxins, total DSP concentrations were higher and total PTX concentrations were lower in blue mussels (*M. edulis*) that were placed for monitoring purposes with OA esters, DTX1 esters, OA and DTX1, representing 49%, 25–27%, 15% and 9–11% of the total, respectively (Figure 4B and Figure 5B). Maximal total DSP concentrations in shellfish coincided with the peak of the *Dinophysis* bloom at site NPB2 (771 ng g^−1^), while the peak at the NPB8 site (485 ng g^−1^) was offset by two weeks (Figure 4A,B and Figure 5A,B). At both sites, mussels first exceeded the USFDA action level (160 ng g^−1^) on 22 May and remained over the closure limit for nearly two months when cell densities were sustained at >1000 cells L^−1^ (Figure 4B and Figure 5B). 

In contrast to both toxicity in the particulate fraction and shellfish, within SPATT samplers, free OA represented the largest proportion (53–61%) of the total DSP toxins followed by DTX1 (20–22%), OA esters (12–18%) and DTX1 esters (5–8%; Figure 4C and Figure 5C). Total PTX concentrations were generally higher than DSP toxins with peak concentrations for sites NPB2 and NPB8 being 1692 and 1052 ng total PTX g^−1^ of dry resin and 327 and 272 ng total DSP g^−1^ of dry resin, respectively (Figure 4C and Figure 5C). The HP20 resins in SPATT samplers detected DSP toxins in the water column (24 April) three-to-four weeks earlier than deployed mussels (15 May; Figure 4B,C and Figure 5B,C). While peak toxicity in HP20 resin was, in some cases, slightly offset compared to mussels, toxin concentrations adsorbed by the HP20 resin were highly and significantly correlated with toxins in blue mussels (*R*^2^ > 0.70, *p* < 0.001), but not *Dinophysis* densities (*R*^2^ = 0, *p* > 0.05) or particulate toxins (*R*^2^ = 0, *p* > 0.05 Figure 4 and Figure 5, Table 1). 

### 3.4. DSP Toxin Analysis: LC/MS vs. PP2A

**Figure 6:**
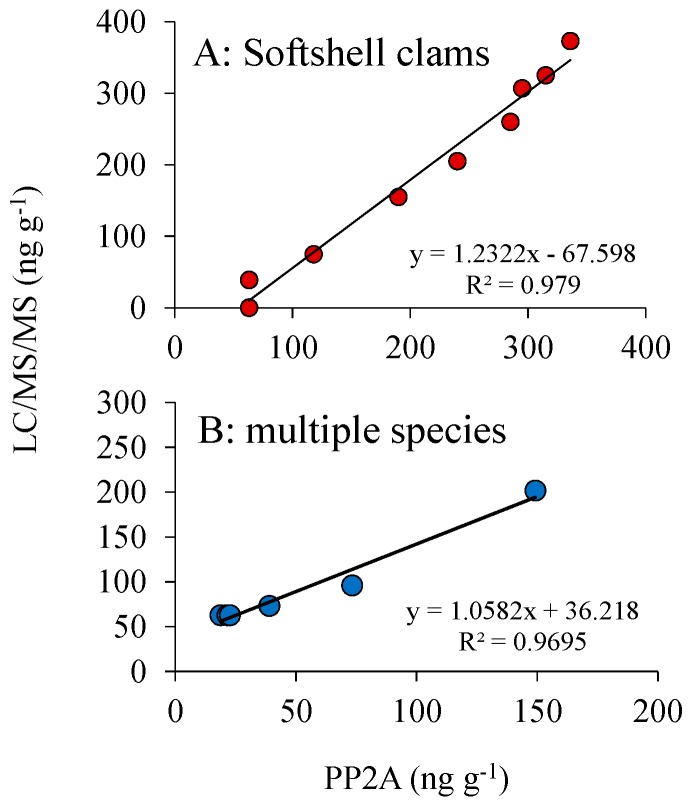
Total DSP toxins (ng g^−1^) in (**A**) softshell clams (*n* = 9) and (**B**) various shellfish species (*n* = 6) from New York embayments analyzed via LCMS and PP2A. Each point represents >6 pooled individuals.

**Figure 7:**
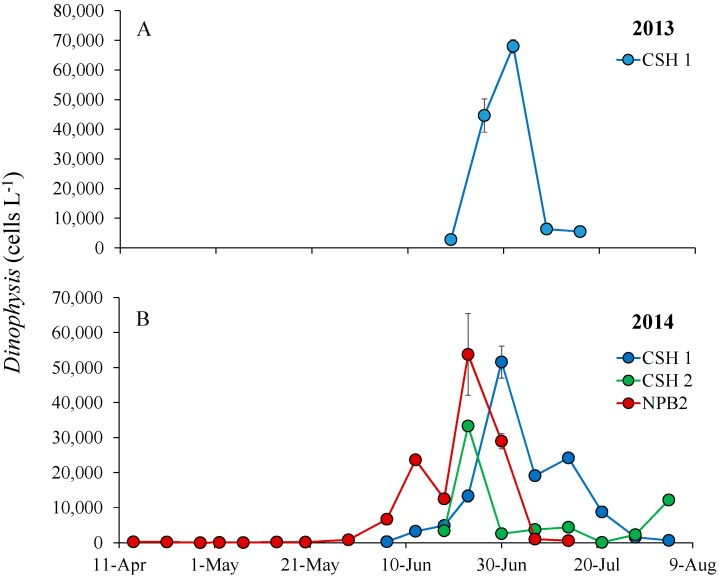
*Dinophysis* densities (cells L^−1^) during the spring and summer of (**A**) 2013 and (**B**) 2014 at Northport Bay (NPB2) and two sites in Cold Spring Harbor (CSH 1 and CSH2). Points are means, while error bars represent the standard deviation of duplicate samples.

The use of the Abraxis protein phosphate inhibition (PP2A) assay was validated using certified reference materials purchased from the National Research Council of Canada, with reference materials including a naturally-contaminated mussel sample (CRM-DSP-mus-c) and a methanolic calibration standard, yielding recoveries between 100% and 113% and a certified non-toxic mussel homogenate (CRM-zero-mus) yielding concentrations below methodological detection limits (Table 2). The double exhaustive and single dispersive extraction methods (measured via PP2A) were used to measure DSP toxins in multiple shellfish samples collected from Northport Bay and Cold Spring Harbor with the double exhaustive extraction method measuring DSP toxins in shellfish ranging from <63–202 ng g^−1^ and the single dispersive extraction method ranging from <63–176 ng g^−1^ (Table 3). Results from both extraction methods measured via PP2A were not significantly (*t*-test; *p* > 0.05) different from each other and were highly correlated (*R*^2^ = 0.99; *p* < 0.001). Shellfish extracts, from various bivalve species, measured via PP2A and LC/MS were not significantly (*t*-test; *p* > 0.05) different from each other and were also highly correlated (*R*^2^ = 0.98; *p* < 0.001; Figure 6, Table 3). Two samples (10 July 2013, blue mussel and 7 July 2014, ribbed mussel) from Cold Spring Harbor were over the federal closure limit (Table 3) after *Dinophysis* sustained densities of 10^4^ cells L^−1^ for >2 weeks (Figure 7). While the 2014 ribbed mussel sample was over the closure limit when measured via PP2A for both extraction methods (202 and 176 ng g^−1^), it was slightly under the closure limit when measured via LC/MS (149 ng g^−1^); however, overall measurements made via PP2A and LC/MS were not significantly different (Table 3). While peak DSP concentrations in mussels were above the USFDA action level (160 ng g^−1^), these mussels originated from a region closed to shellfishing due to the presence of high levels of coliform bacteria.

During 2013 and 2014, *Dinophysis* densities in Cold Spring Harbor and Northport Harbor approached 10^4^ cells L^−1^ in late June and early July (Figure 7). Oysters (*Crassostrea virginica*) and clams (*Mya arenaria*) sampled the week after the peak (~70,000 cells L^−1^; Figure 7) of the 2013 Cold Spring Harbor bloom, when densities had dropped an order of magnitude (~6000 cells L^−1^; Figure 7), had 25 and 78 ng g^−1^ of total DSP toxins as measured via LC/MS (Table 3). In Northport Bay 2014, despite sustained densities of 10^4^ cells L^−1^ for four weeks (Figure 7), shellfish collected on 2 July 2014 were below the detection limit (<63 ng g^−1^) for the PP2A and were <25 ng g^−1^ as measured via LC/MS (Table 3). 

## 4. Discussion

This study explored differing approaches for monitoring DSP and PSP. By monitoring the dynamics of pelagic *Alexandrium* and *Dinophysis* cells, pelagic toxins, toxins in shellfish and toxins in SPATT samplers with two types of resin, HP20 and XAD-2, and comparing extraction techniques and analytical approaches for toxin analysis, a comprehensive assessment of methodologies was generated. While SPATT samplers coupled with PP2A technology proved to be highly efficient for monitoring the dynamics of DSP, SPATT did not provide data useful for the monitoring of PSP. Collectively, this study provides new insight useful for developing effective biotoxin monitoring programs in coastal ecosystems. 

### 4.1. DSP SPATT Samplers 

To date, several studies have explored the use of SPATT technology to track saxitoxin, microcystin and domoic acid in North America [23,24,25,51]. While the use of SPATT for monitoring DSP toxins has been widely explored in Europe and New Zealand [22,27,28,29], the use of SPATT for DSP monitoring has not been investigated in North America despite the recent emergence of DSP events on the East, West and Gulf Coasts of this continent [12,13,14,15,16,17]. In the present study, HP20 adsorbed ~3-times more DSP toxin than XAD-2 resin. While XAD-2 has not been utilized as a toxin tracking resin for DSP in prior studies, HP20 has and, in agreement with our findings, has been found to have a high adsorption affinity for DSP toxins [22,27,28]. Further, consistent with prior studies [22,27,28,29], HP20 was capable of detecting multiple DSP toxins, including OA, DTX1 and their esters, all of which are considered in regulatory closure limits. Several PTX congeners were also detected using HP20 resin [22,27,28], which are considered monitored and regulated in the EU [52], but not the U.S. [35]. This study demonstrates that SPATT samplers containing HP20 resin comprise a promising HAB monitoring technology for the U.S. East Coast, which is easy, safe and forgoes the use of live animals (mussels) hung for monitoring purposes that are time consuming in their weekly maintenance and extraction. Moreover, its highly sensitive nature and ability to adsorb DSP toxins more than three weeks prior to their detection in shellfish makes them an ideal candidate as a sentinel or early warning system for the emergence of DSP within ecosystems with active shellfish harvesting. While SPATT is not a replacement for shellfish testing, its utility as an early warning system could guide shellfish testing efforts and aid in resource allocation for toxin monitoring programs.

### 4.2. DSP in SPATT, Bivalves and Cells 

SPATT was capable of tracking *Dinophysis* densities and DSP toxins in phytoplankton concentrates and shellfish. Similarly, MacKenzie et al. [22] found good linear relationships (*R*^2^ > 87) between total DSP toxins in shellfish (greenshell and blue mussels) and SPATT. This pattern was clear for both the Britannia and Woodbine sites of Northport Harbor during the present study, but these relationships were not always statistically significant (Table 1). In fact, toxins in SPATT predicted shellfish toxicity better than particulate toxins or *Dinophysis* densities (Table 1). For example, while toxins in HP20 resin were highly and significantly correlated (*R*^2^ = 0.89 and 0.70, *p* < 0.001, for Britannia and Woodbine, respectively) with total DSP toxins in shellfish, the relationship between toxins in phytoplankton concentrates and toxins in resins was non-existent (*R*^2^ < 0; Table 1). This may, in part, be due to peak total DSP toxins in resins occurring a week later than the peak of the bloom, which may represent increased extracellular excretion by *Dinophysis* cells due to bloom demise and cellular stress [53]. Alternatively, other factors such as the hydrodynamics of the study sites or the inability to detect toxins at low *Dinophysis* densities may be responsible for the lack of a statistical relationship between toxins in phytoplankton concentrates and toxins in resins. Another pattern observed during this study, was the difference in toxin composition amongst the toxins found in cells, shellfish and HP20 resin, which were consistent among the two study sites. This difference is not surprising for shellfish as toxins undergo biotransformations with the tissue [22,25,28]. Smith et al. [53] found that the release of extracellular toxins from another strain of *Dinophysis acuminata* are a stress/death response. The differences seen between the toxins in cells compared to the resin may reflect a contrast between what is excreted by cells and what is retained intracellularly as Nagai et al. [54] demonstrated the release of extracellular toxins in cultures of *Dinophysis* acuminata. Alternatively, differences could also reflect the transformation of these toxins within the water column via bacterial degradation (as seen for other toxins; [55,56]). While this study found that SPATT samplers were able to detect toxins in the water column 3–4 weeks earlier than shellfish detections and MacKenzie et al. [22] reported SPATT DSP detection at least a week earlier than shellfish, Pizarro et al. [28] found that *Dinophysis* cells provided a more reliable early warning of shellfish toxicity than SPATT. Regardless, the ability of SPATT resins to track *Dinophysis* densities, as well as to mimic shellfish toxicity makes this a highly promising technology for DSP monitoring programs. 

The Abraxis protein phosphate inhibition assay (PP2A) was found to be an excellent candidate for integration into monitoring programs due to its correlation with LC/MS data, ease of use and rapid results (within a day). In this study, PP2A was capable of detecting DSP toxins in certified reference methanolic standards and shellfish obtained from NRC, as well as shellfish collected from shellfish beds in New York. In addition, while DSP toxin concentrations using the double-exhaustive extraction method [50] were not statistically significantly different from those of the single dispersive extraction method, it yielded slightly higher toxin concentrations. While ELISA assays have similar potential as PP2As, given the DSP toxin composition in shellfish (and *Dinophysis*) in the NY region, the Abraxis ELISA DSP assay would be expected to significantly underestimate toxin concentrations in shellfish given that there was only 50% cross reactivity with one of the dominant congeners, DTX1 (>30% of total toxicity). This highlights the importance of prescreening shellfish using LC/MS to assess congener composition for newly-studied areas to determine the most efficient and appropriate analytical method to explore for monitoring programs.

### 4.3. PSP SPATT Samplers and Toxicity 

While the use of SPATT samplers containing HP20 resin is a highly promising monitoring technology for DSP toxins, data collected during this project demonstrated that SPATT samplers were not an optimal choice for PSP monitoring in this region. Despite *Alexandrium* being capable of releasing extracellular toxins into the water column [33], measurable levels of PSP toxins from HP20 and XAD-2 resins were detected on only three occasions and did not track *Alexandrium* densities or toxicity in shellfish. In contrast, Lane et al. [25] found that HP20 extracts reflected saxitoxin levels measured in sentinel mussels and *Alexandrium* densities in coastal California. Using different resins (CDP and SP700), Rodríguez et al. [26] demonstrated that the resins CDP and SP700 adsorbed only 1% and 4–20% of paralytic shellfish toxins. Further, rapid toxin desorption was found for both of these resins with differential desorption for the C toxins, GTXs and the neoSTX and STX toxins [26], suggesting that the efficacy of resins in general may vary with the toxin composition of different *Alexandrium* strains. While the resins used in this study differed from those of Rodríguez et al. [26], it is possible that the toxin composition of the NY strain is not compatible with certain resins given that PSP toxins in this region are dominated by C toxins and GTXs [57,58]. Currently, there are no assessments of differential desorption of PSP congeners from HP20 or XAD-2 resins. Alternatively, the NY *Alexandrium* strain may not excrete sufficient concentrations of toxin to be adsorbed and subsequently detected using these resins. Regardless, differences among these studies demonstrate the need for region-specific assessments of resins for use with SPATT monitoring. 

While there was no coherence between the PSP toxins found in SPATT resins and shellfish toxicity, shellfish toxicity did mirror *Alexandrium* densities and particulate saxitoxin concentrations. Comparing analytical methodologies, in this study, the receptor binding assay (RBA) yielded higher PSP toxin concentrations in cell pellets than measurements via LC/MS. Further, PSP toxin levels quantified via LC/MS were lower than MBA estimates. This is likely due, in part, to the MBA estimation of total toxicity in the sample, compared with the LC/MS detection of individual toxin congeners.

### 4.4. The Co-Occurrence of DSP and PSP Toxins in Shellfish 

The co-occurrence of *Alexandrium* and *Dinophysis* blooms and their toxins in the water column has been previously demonstrated in the New York region [14], and this study further demonstrated that these toxins also co-occur in shellfish (Figure 3 and Figure 5). Saxitoxin, okadaic acid, dinophysistoxin and pectenotoxins were all detected in blue mussels deployed in Northport Bay (NPB8), New York. Moreover, both PSP (saxitoxin) and DSP (okadaic acid and dinophysistoxins) toxins co-occurred in concentrations that were over the USFDA action levels. Similarly, the co-occurrence of DSP and PSP-toxins in shellfish have been reported in Europe [59] and South America [60]. Within the past decade, there have been other reports of marine toxins such as saxitoxin and domoic acid, co-occurring in planktivorous fish, shellfish and even marine mammals [61,62]. The presence of multiple HAB toxins in shellfish is an emerging issue that warrants further investigation given the unknown additive or synergistic effects of these toxins on human health and marine organisms.

## 5. Conclusions

In a recent review [7], it was reported that marine biotoxin-contaminated seafood has caused thousands of poisonings worldwide during the first 15 years of the 21st Century. Given that ocean warming has, and may continue to, exacerbate PSP and DSP events globally [63], technologies that fast-track shellfish bed closures to protect human health are needed. Overall, this study provides information that can help facilitate the implementation and integration of important, new technologies into coastal monitoring programs. 

## Figures and Tables

**Table 1:** Linear regressions for particulate toxin (pg mL^−1^), *Dinophysis* densities (cells L^−1^), total DSP toxins in shellfish (ng g^−1^) and total DSP toxins in SPATT (ng g^−1^ of dry resin); *R*^2^ and *p*-value are reported. Significant correlations are bolded.

NPB2B	Particulate DSP Toxin	*Dinophysis* Densities	DSP Toxins in Shellfish
Particulate DSP toxin	-	-	-
*Dinophysis* densities	0.99 (*p* < 0.001, *n* = 26)	-	-
DSP toxins in shellfish	0.35 (*p* > 0.05, *n* = 9)	0.38 (*p* < 0.05, *n* = 9)	-
DSP toxins in SPATT	0 (*p* > 0.05, *n* = 11)	0 (*p* > 0.05, *n* = 11)	0.89 (*p* < 0.001, *n* = 8)
NPB8B			
Particulate DSP toxin	-	-	-
*Dinophysis* densities	0.99 (*p* < 0.001, *n* = 10)	-	-
DSP toxins in shellfish	0.05 (*p* > 0.05, *n* = 8)	0.18 (*p* > 0.05, *n* = 12)	-
DSP toxins in SPATT	0 (*p* > 0.05, *n* = 8)	0 (*p* > 0.05, *n* = 12)	0.70 (*p* < 0.001, *n* = 11)

**Table 2:** Recovery of certified reference materials from National Research Council of Canada used in multiple analyses to validate protein phosphate inhibition (PP2A) assay kit purchased from Abraxis. CRM, certified reference material; mus-c, contaminated mussel.

Sample ID	Total OA	Recovery
CRM-OA-c	1.7 ± 0.1 nM	113%
CRM-DSP-mus-c 1	3003 ± 9.3 ng g^−1^	100%
CRM-DSP-mus-c 2	3013 ± 183 ng g^−1^	100%
CRM-DSP-mus-c 3	3159 ± 191 ng g^−1^	105%
CRM-zero-mus 1	<63 ng g^−1^	
CRM-zero-mus 2	<63 ng g^−1^	
CRM-zero-mus 3	<63 ng g^−1^	

**Table 3:** Total DSP toxins (ng g^−1^) in wild shellfish harvested from regions of New York. Comparison of two extraction methods (double exhaustive and single dispersive) and comparison of two analytical methods (protein phosphatase inhibition assay, PP2A; and liquid chromatography mass spectrometry, LC/MS). Bold indicates over the federal closure limit (160 ng g^−1^).

Bay	Site	Date	Species	Total DSP ng g^−1^		
				PP2A		LCMS
				Double Exhaustive	Single Dispersive	Double Exhaustive
Cold Spring Harbor	**CSH1**	**7/10/2013**	***Mytilus edulis***			**168**
	CSH1	7/10/2013	*Crassostrea virginica*			25
	CSH1	7/10/2013	*Mya arenaria*			78
	**CSH1**	**7/7/2014**	***Geukensia demissa***	**202**	**176**	**149**
	CSH1	7/14/2014	*Geukensia demissa*	73	69	39
	CSH1	7/21/2014	*Mytilus edulis*	96	90	73
	CSH1	7/21/2014	*Geukensia demissa*	<63		23
	CSH2	7/7/2014	*Mytilus edulis*	81		
	CSH2	7/14/2014	*Mytilus edulis*	89		
Northport Bay	SB	7/2/2014	*Geukensia demissa*	<63		19
	SB	7/2/2014	*Mya arenaria*	<63	<63	22
